# The Effect Citrox BCL on *Legionella pneumophila* Mechanisms of Biofilm Formation, Oxidative Stress and Virulence

**DOI:** 10.3390/antiox11112186

**Published:** 2022-11-04

**Authors:** Eugenia Butucel, Igori Balta, David McCleery, Cosmin Alin Popescu, Tiberiu Iancu, Ioan Pet, Adela Marcu, Nicolae-Marinel Horablaga, Lavinia Stef, Nicolae Corcionivoschi

**Affiliations:** 1 Bacteriology Branch, Veterinary Sciences Division, Agri-Food and Biosciences Institute, Belfast BT4 3SD, UK; 2 Faculty of Bioengineering of Animal Resources, University of Life Sciences King Mihai I from Timisoara, 300645 Timisoara, Romania; 3 Faculty of Agriculture, University of Life Sciences King Mihai I from Timisoara, 300645 Timisoara, Romania; 4 Faculty of Management and Rural Development, University of Life Sciences King Mihai I from Timisoara, 300645 Timisoara, Romania

**Keywords:** *L. pneumophila*, Citrox BCL, biofilm, virulence, siderophores

## Abstract

*Legionella pneumophila* is responsible for causing Legionnaires’ disease and Pontiac fever, also known as legionellosis. The aim of this study was to investigate the mechanistic effect of a mixture of natural antimicrobials (Citrox BCL) in preventing *L. pneumophila* biofilm formation and reducing its in vitro virulence. The minimum inhibitory concentrations were detected at 0.06%, and the MBC was established at 0.125%. Based on the growth curve profile, the sub-inhibitory concentration of 0.02% was further used to study the mechanistic implications in the absence of a cytotoxic effect on A549 cells. At 24 h post-infection, Citrox BCL reduced (*p* = 0.005) the intracellular growth of *L. pneumophila* when the A549 cells or the bacteria were pre-treated with 0.02% Citrox BCL. This result was replicated when Citrox BCL was added during the 24 h infection assay leading to a reduction in intracellular growth (*p* = 0.003). Herein we show that at the sub-inhibitory concentration of 0.02%, Citrox CBL lowers the ROS levels in infected A549 cells and causes a 45% reduction in *L. pneumophila* EPS production, a reduction associated with the decline in biofilm formation. Overall, our results corroborate the low c-di-GMP production with the decrease in biofilm formation and low EPS levels. The low EPS levels seemed to be caused by the downregulation of the *tat*B and *tat*C gene expressions. Moreover, inhibition of *pvc*A and *pvc*B gene expressions, leading to lower siderophore levels, suggests that Citrox BCL reduces the ability of *L. pneumophila* to sequester iron and reduce biofilm formation through iron starvation.

## 1. Introduction

The discovery of Legionnaires’ Disease began in 1968 in Pontiac, Michigan, USA, which was named *Legionellosis* in the aftermath [1]. Later in 1976, the first unprecedented outbreak of pneumonia was registered at the 58th Annual American Legion Convention from a hotel localised in the state of Philadelphia, where 221 people faced clinical criteria for acute respiratory syndrome, of which 34 died as a result [2,3]. *Legionella* species (spp.) are gram-negative bacillus pathogens included in the gammaproteobacterium class, a genus widely dispersed in nature, regularly identified soil, with the most frequent isolation rate in fresh surface waters and drinking water environments [1]. Currently, there are more than 50 *Legionella* spp. and above 60 different serogroups, whereas *Legionella pneumophila* serogroup 1 is reported as the most virulent species responsible for ≈85% of the documented cases, while *Legionella longbeachae* and *Legionella bozemanii* are responsible for 3.9% and 2.4% of cases [1,4].

A unique trait of this pathogen is to thrive in plumbing systems by forming biofilms by employing protozoa hosts to protect *legionella* against various disinfectants, biocides, and environmental stressors. This pathogen adopts an intracellular biphasic lifestyle and replicates inside the alveolar macrophages or in the interior of the multiple protozoa (*Tetrahymena*) hosts, including amoebae (e.g., *Acanthamoeba*, *Vermamoeba*, and *Hartmanella*) [5,6]. The lifestyle of being a member of parasitising free-living amoeba or nematodes and a part of multispecies biofilm are only a few biological factors that aid *L. pneumophila* biofilm formation [7]. *L. pneumophila* could overwhelm nutrient limitations from environmental settings by establishing multispecies biofilms. Consequently, *L. pneumophila* adheres to a pre-installed biofilm by other bacteria rather than adhering to the surfaces as the first colonising agent, which assists in increasing the pathogen survivability by incorporating it in the biofilm assembly [8]. The presence of the *Lqs* system in *L. pneumophila* is responsible for regulating biofilm formation. *L. pneumophila* in biofilms has increased resistance toward different biocidal agents, and there were cases when Legionellosis outbreaks were previously ascribed to established biofilms [7].

Over the last four decades, examining *Legionella* has opened new insights into its virulence strategies and mechanisms used by this bacterium to replicate between mammalian phagocytes and other differential environments that later trigger human infection. *Legionella* is reported to comprise the largest and most different effector repertoire among other intracellular pathogens [9]. Hence identification of novel strategies to prevent *Legionella* biofilm formation on various surfaces becomes of major importance for both animal and human health. Combinations of soluble bioflavonoids (Citrox) obtained from citrus fruits demonstrated antimicrobial activity against bacterial species and candidal species tested at a concentration of 1% (v/v) in both the broth and the biofilm assay [10]. The addition of 1–2% Citrox and 1% chitosan to meat samples lead to a 4-log reduction of C. jejuni; hence, combinations of antimicrobials show a great potential to increase meat safety and preservation [11]. Hence, searching for natural alternatives to disinfect water systems is attracting even more attention. Mixtures of antimicrobials, plant extracts, essential oils, organic acids and antimicrobial peptides have an important role in research for drug and antimicrobial substances development and are currently documented in many studies regarding safe biological effects and effectiveness against a wide range of pathogens [12,13,14,15]. There are multiple examples of natural antimicrobials with an anti-biofilm role, including the essential oil extracted from lemon (*Citrus Limonum*), which displayed increased biofilm inhibitory capability above 63% and biofilm eradication values by approximately 34.2% at the concentration of MIC/2 [15]. The same author also showed that sage (*Salvia officinalis*) and thyme (*Thymus vulgaris*), essential oils at their MICs, resulted in significant biofilm eradication and displayed values close to 39 and 31%, respectively. Other studies suggested that essential oils containing α, β-pinene and D-limonene are speculated to evoke efficiency for controlling *L. pneumophila* [16,17]. For example, α-pinene at 200 mg/mL concentration during disk diffusion assay produced huge zones of inhibition by representing zones of 70 mm against *L. pneumophila* (strain 130b) [17]. Similarly, at the same concentrations, essential oils from niaouli (*Melaleuca quinquenervia*), immortelle (*Helichrysum italicum*), rosemary (*Rosmarinus officinalis*) and juniper berry (*Juniperus communis*) produced notable inhibitory zones ranging from 33.3 to 55.5 mm.

Combinations of organic acids with citrus extracts have become more accessible as antimicrobial compounds (in mixture) due to the recently published data describing their biological mode of action both in vitro and in vivo [18,19]; however, little or no information is available regarding their effect on the virulence mechanisms of *L. pneumophila*. The aim of this study was to investigate the mechanistic effect of Citrox BCL in preventing not only in vitro infection of epithelial cells but also to describe its effect against biofilm formation.

## 2. Materials and Methods

### 2.1. Determination of Minimum Inhibitory and Minimum Bactericidal Concentration

The two-fold tube dilution method was used to determine the lowest concentration of Citrox BCL that inhibited bacterial growth (MIC), and the lowest concentration that induced bacterial death (MBC) was evaluated. Citrox BCL is trial formulation obtained from Citrox Biosciences UK. The technical data sheet provided detailed formulation (lactic acid 20%, caprylic acid 10%, citric acid 5% and 5% malic acid. The remaining 60% is citrus extract, glycerine, surfactants and water. Citrox BCL was diluted (8% to 0.015625% v/v) in Yeast Extract BYCE Broth and thoroughly vortexed. Overnight bacterial culture was harvested by centrifugation, washed twice in PBS, re-suspended in BYCE Broth, and diluted to 1 × 10^6^ CFU/mL in BYCE Broth. Each tube was inoculated with 5 × 10^5^ CFU/mL bacterial culture (final concentration). Non-inoculated bijou (5 mL) tubes containing the same growth medium were used as negative controls, whilst BYCE Broth tubes without Citrox BCL were inoculated with bacterial culture as positive controls. Tubes that did not show visible growth were above the MIC. The highest dilution of each antimicrobial with no microbial growth was considered as the MBC. The antimicrobial mixture was tested using concentrations from 0–0.08 to 0.0078% (v/v) in three independent replicates repeated three times for each strain. In order to determine the sub-inhibitory concentrations used, the pathogen was exposed to different concentrations of the antimicrobial mixture. The highest concentrations of antimicrobial that showed no effect on survivability and no growth inhibition (same growth kinetics as the control) were used in subsequent experiments.

### 2.2. Infection and Cytotoxicity Assay

The strain of *L. pneumophila* SG68703154 was grown in Yeast Extract BYCE Broth at 35 °C in a humidified incubator as previously described (laboratory isolate). The human alveolar epithelial cell line A549 (ATCC-CLL185) was maintained in DMEM media (Sigma-Aldrich, (Sigma-Aldrich, Gillingham, England, UK) containing 10% heat-inactivated fetal bovine serum (Sigma-Aldrich, Gillingham, England, UK), supplemented with 0.5% penicillin and streptomycin at 37 °C in humidified air under 5% CO_2_. The infection assay with A549 cells was performed in 24-well plates containing 10^5^ cells/well subsequently infected with pre-treated (0.02% Citrox BCL) or untreated bacteria at a multiplicity of infection of 100 (MOI 100). The infected cells were centrifuged at 1500 rpm for 10 min. After incubation for 2 h, the extracellular fluid and bacteria were removed by washing 3 times with tissue culture medium, and the plates were further incubated for up to 3 days. At various times, the cultured cells were desquamated into the supernatant by gentle scratching with a pipette tip. The supernatant was finally harvested and diluted appropriately with sterile distilled water and subsequently cultured on BCYE-α agar. The cytotoxicity of Citrox BCL was determined as previously described [20] using the MTT assay (Sigma-Aldrich, Gillingham, England, UK). 

### 2.3. c-di-GMP Assay

The cytosolic c-di-GMP measurement was performed as previously described [21]. Briefly, they were grown in 24-well plate, in the presence of 0.02% Citrox BCL, and collected for up to 24 h. Moreover, a co-culture experiment of *L. pneumophila* SG68703154 with A549 cells and 0.02% Citrox BCL was set up and supernatant bacteria were harvested by centrifugation at 13,000 g after 24 h. Samples were collected for analysis at 3, 9, 15 and 24 h. The centrifugation was performed at 13,000 g for 2 min and at 4 °C. Cell samples were used to determine total protein concentration using a Quick Start Bradford 1×dye reagent (Brennan, Ireland). The liquid supernatant was used to measure the intracellular c-di-GMP concentration1 using a Cyclic di-GMP ELISA Kit (Thermo-Fisher, Loughborough, UK). The intracellular c-di-GMP concentrations were converted to pmol/mg of protein. The experiment was performed in triplicate and on three separate occasions.

### 2.4. Biofilm Microtiter Plate Assay

Overnight cultured *L. pneumophila* SG68703154 (100 μL, OD_600_~0.2) was used to inoculate 24 well plates containing Yeast Extract BYCE Broth, followed by incubation at 35 °C in a humidified incubator in the presence of 0.02% Citrox BCL. The 24-well plates were incubated at 35 °C for 24 h without shaking. To quantify biofilm formation, wells were washed three times with PBS to remove all non-adherent cells. Cells were fixed with 200 µL of methanol for 20 min. Biofilms were stained with crystal violet (0.4% w.v) for 15 min and washed three times with PBS. The formed biofilm was then dissolved with 200 µL 96% ethanol for 30 min, and absorbance was measured (570 nm) in a microtiter plate reader. Cultures with no added antimicrobial served as positive controls.

### 2.5. RNA Extraction and qRT-PCR

This assay was designed to measure *tet*B and *tet*C virulence genes expression in *L. pneumophila* SG68703154 exposed or grown in the presence of Citrox BCL. Total RNA was isolated from bacteria exposed to 0.02% Citrox BCL for 24 h by using the RNeasy Plus Mini Kit (Qiagen, Manchester, UK). The RNA was reverse transcribed using Transcriptor First Strand cDNA Synthesis Kit (Roche, Dublin, Ireland) according to the manufacturer’s protocol. The mRNA levels were determined by quantitative RT-PCR using QuantiNovaSYBR^®^ Green PCR Kit (Qiagen, Manchester, UK) on a LightCycler 96 (Roche, Dublin, Ireland). The primers used (Thermo-Fisher, Loughborough, UK) were 5′-tgacactcaaaaagacg-3′ and 5′-tgctaatattcaatcgg-3′ for *tat*B and 5′-taacccggcttgaattg-3′ and 5′-atgctgagttgccctgg-3′ for *tat*C [22]. For *pvc*A, the primers used were F: gtgtcccggaaaccattagt and R: cgggaaggcaggcaatataa with the probe FAM-aaagtgcgcttcacctcatttgcc-IBFQ, and for *pvc*B, the primers were F: agcgttacagtttgcctctt and R: ggtgataccgtcttgctgttat with the probe FAM-aggtgagtggtacctaccaaagga-IBFQ. For probe assay, the 20 μL PCR mix comprised 4 μL of 82 cDNA, 1× PrimeTime Gene Expression Mastermix (IDT), and 1 μL of 20× Probe assay mix (4 μM probe and 10 μM of each primer) [23]. The conditions for genes rRNA 16S consisted of incubating for 10 min at 95 °C followed by 45 cycles of 95 °C for 10 s, 55 °C for 30 s, and 72 °C for 10 s. A total of 5 μL of SYBR Green master mixture was used in each reaction along with 0.5 μL of 10 μM primer mixture, 3 μL of molecular grade water, and 1 μL of DNA sample. The 2^–DDCT^ method was used to analyse the relative expression (fold changes), calculated relative to the control group. The expression of GAPDH gene was used as a control. Quantification of *L. pneumophila* in biofilm was performed as previously described [24]. Briefly, after biofilm formation in the presence or absence of 0.02% Citrox BCL, the adherent cells were removed, and DNA was extracted using a commercial DNA extraction kit (PureLink, Invitrogen, UK). For the quantitative PCR assays, specific primers for *L. pneumophila*, Mip A1 (5ʹ-gcattggtgccgatttgg-3ʹ) and Mip A2 (5ʹ-gytttgccatcaaatctttctgaa-3ʹ), were used. A positive genomic DNA control and water, as negative control, were included in all PCR assays. A standard curve was obtained with 10-fold serial dilutions of a known amount of *L. pneumophila* SG68703754 genomic DNA. All results are expressed in Genome Units (GU).

### 2.6. Exopolysaccharide (EPS) Measurement and Motility Assay

EPS measurement was performed as previously described [25]. Briefly, an *L. pneumophila* culture grown with and without 0.02% Citrox BCL was centrifuged, and the supernatant was filtered through a 0.22 µm filter. Three volumes of chilled 100% ethanol were added to the filtered supernatant and incubated overnight at 2 °C to precipitate the EPS, and after the ethanol wash, EPS was quantified using the colorimetric phenol-sulphuric acid method. To analyse the effect of Citrox BCL on *L. pneumophila* culture 10 μL of early stationary phase cultures of *L. pneumophila* culture grown for 24 h in the presence of 0.06% Citrox BCL were dropped onto fresh BCYE plates containing 1.6% agar [26]. As a control, 24 h bacterial growth in the absence of Citrox BCL was used. The inoculated plates were incubated at 30 °C, and growth was observed for the next 48 h. Motility was assessed as the measurement from the centre inoculation spot to the edge of growth circle and expressed in millimetres (mm). Experiments were performed in triplicates and on three separate occasions. 

### 2.7. Intracellular ROS in L. pneumophila SG68703154 Infected A549 Cells

The production of intracellular ROS was measured using 2′,7′-dichlorofluorescein diacetate (DCFH-DA) as previously described [27]. Briefly, a 10 mM DCFH-DA stock solution (in methanol) was diluted 500-fold in PBS to obtain a 20 μM working solution. After *L. pneumophila* infection, with or without 0.02% Citrox-BCL, the cells in a 12-well plate were washed twice with PBS and then incubated in a 100-μL working solution of DCFH-DA at 37 °C for 30 min. Fluorescence was then determined with an excitation wavelength of 485 nm and an emission wavelength of 520 nm using a microplate reader (FLUOstar Omega from Premier Scientific, Belfast, UK). The ROS levels are expressed as fold change over the infected and treated control.

### 2.8. Siderophore and Intracellular Iron Measurement

Siderophore activity was measured in culture supernatants in the presence or absence of 0.02% Citrox BCL. The method used was previously described [28]. Briefly, bacteria were grown in BYE broth in the presence of 0.02 Citrox BCL for 24 h. After 24 h, 3 mL of supernatant was harvested and used to test for siderophore activity using the CAS assay [29], expressing the siderophore activity as equivalents of desferrioxamine (DFX) [30]. The intracellular iron concentration in *L. pneumophila* biofilms in the presence or absence of 0.02% Citrox BCL was measured as described [31,32]. Iron levels were normalised with the protein concentrations determined by the Bradford assay. All experiments were conducted in triplicate and at three separate times.

## 3. Results

### 3.1. Citrox BCL Effect on L. pneumophilia Growth, Minimum Inhibitory Concentration (MIC) and Minimum Bactericidal Concentration (MBC)

**Figure 1:**
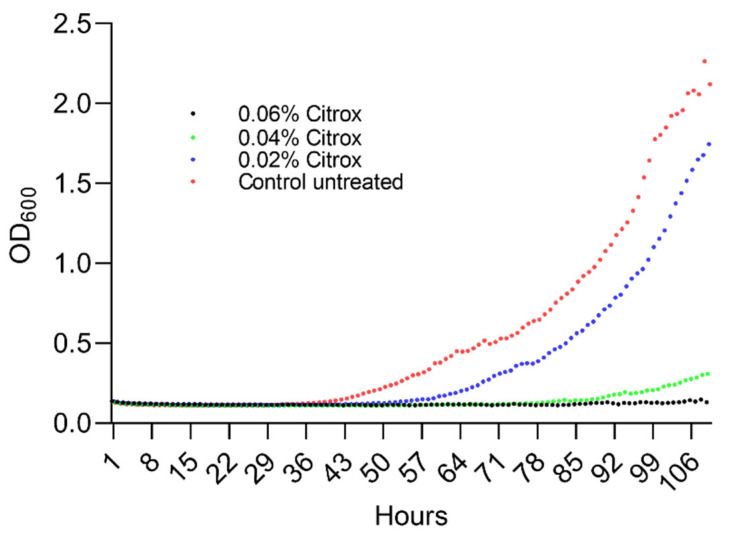
The impact of Citrox CBL on growth and survival of *L. pneumophila* at concentrations of 0.02–0.06%. The MIC concentrations tested are indicated on the graphs in order to establish the sub-inhibitory concentration for further investigations. The experiments were performed in triplicate and on three separate occasions. In order to quantify the growth, the absorbance was measured at 600 nm every 3 h for 36 h.

First, we had to identify the sub-inhibitory concentrations (MIC and MBC) of Citrox BCL and investigate the effect of Citrox BCL in modifying and attenuating the virulence of *L. pneumophila*. The minimum inhibitory concentration was established at 0.06%, and the MBC was determined at 0.125%. Following the analysis of *L. pneumophila* growth curves, complete inhibition of growth at MBC and MIC, as expected; however, the 1/2 MIC (0.25% v/v) concentrations of 0.04% caused a prolongation of the lag phase, whereas 0.02% showed a very similar growth curve to the control indicating no significant growth defect (Figure 1). Therefore, the sub-inhibitory concentration of 0.02% was selected to further study the effect on biofilm formation and in vitro virulence.

### 3.2. Citrox BCL Reduces L. pneumophila Infection of A549 Cells

**Figure 2:**
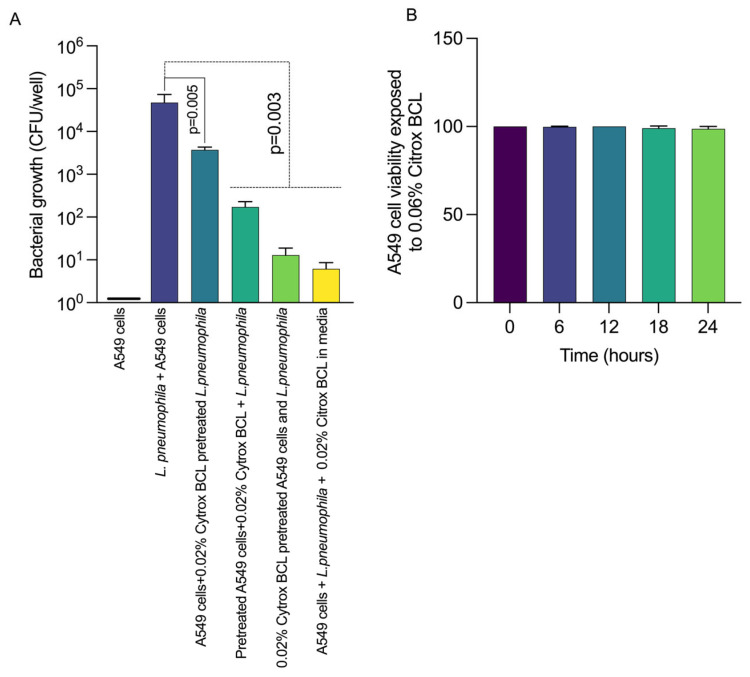
*In vitro* effect of 0.02% Citrox BCL on the internalisation of *L. pneumophila* in A549 cells Panel (**A**) and cytotoxicity Panel (**B**). Results are presented as CFU/well of three biological replicates, and the *p* values are indicated on graphs.

Initially, we aimed to verify if the sub-inhibitory concentration of 0.02% Citrox BCL can inhibit the invasion of A549 cells by *L. pneumophila*. After 24 h of infection, we observed (Figure 2, panel A) that Citrox BCL was able to significantly reduce (*p* = 0.005) the intracellular presence of *L. pneumophila* when the A549 cells or the bacteria were pre-treated with 0.02% Citrox BCL. A similar significant result was observed when Citrox BCL was present in the media during the 24 h infection assay leading to a reduction in the intracellular presence (*p* = 0.003). To determine the cytotoxic effect of Citrox BCL in A549 cells, we measured LDH levels in the supernatants (Figure 2, panel B), indicating no cytotoxic effect. Taken together, these results show that Citrox BCL can reduce the invasion of *L. pneumophila*, an effect believed to be achieved by having a dual effect on the A549 cells and the bacterium itself. 

### 3.3. Citrox BCL Mediates Biofilm Formation and tetB and tetC Biofilm Related Gene Expression during Infection

**Figure 3:**
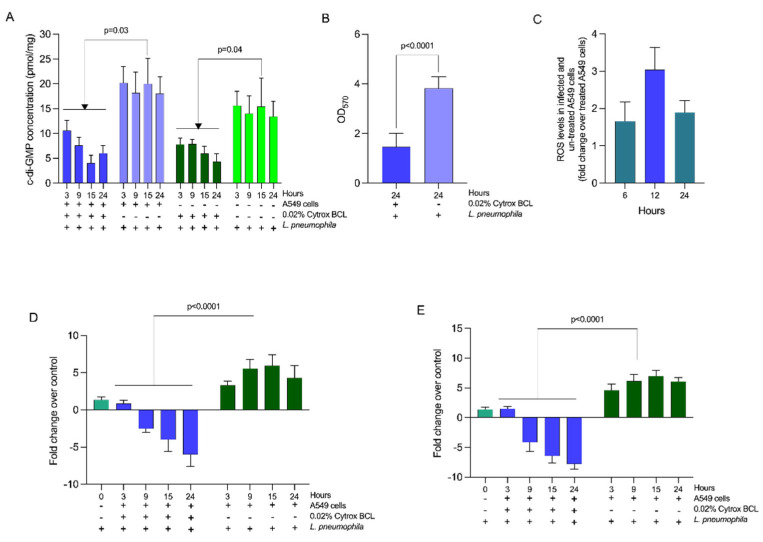
Intracellular concentration of c-di-GMP levels, biofilm formation and virulence gene expression. Panel (**A**)—c-di-GMP levels; Panel (**B**)—biofilm biomass; Panel (**C**)—ROS levels in infected and un-treated A549 cells; Panels (**D**,**E**)—transcriptional analysis of *tat*B and *tat*C. Data are presented as the mean ± SD. Student’s *t*-test was used to analyse the statistical significance as indicated on the graphs. All experiments were performed in triplicate and on three separate occasions.

We have next investigated the biological mechanisms involved by Citrox BCL to reduce the infection of A549 cells by *L. pneumophila*. Our hypothesis, suggesting that the levels of cyclic-imeric diguanylate (c-di-GMP) messenger in *L. pneumophila* are lowered in the presence of Citrox BCL, was confirmed (Figure 3, panel A) and restored in its absence at significant levels (*p* = 0.03). We have also measured the c-di-GMP levels in a co-culture scenario with A549 cells with similar results (Figure 3, panel A), suggesting that Citrox BCL directly acts upon *L. pneumophila* without A549 cell mediation (*p* = 0.04). Moreover, we show that the decreased levels of c-di-GMP levels were also accompanied by significantly lower (*p* < 0.0001) levels of biofilm formed after 24 h in the presence of 0.02% Citrox BCL (Figure 3, panel B). The high levels of bacterial c-di-GMP in the absence of Citrox-BCL were also associated with increased levels of ROS in infected A549 cells (Figure 3, panel C). We have also assumed that the above-observed reduction in virulence and biofilm formation is also accompanied by decreased expression of two of the major virulence genes, *tat*B and *tat*C. Our results show that the expression of both these genes was significantly reduced (*p* < 0.0001) in a time-dependent manner from 3–24 h (Figure 3, panels D and E).

### 3.4. Impact on EPS Production and Bacterial Motility

**Figure 4:**
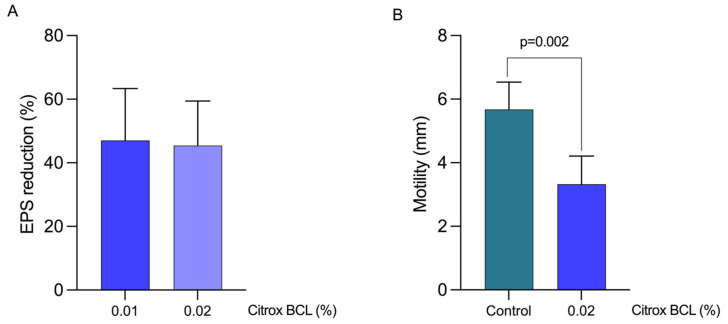
Determination of EPS inhibition of *L. pneumophila* after exposure to 0.01–0.02% Citrox BCL for EPS (panel (**A**)) and 0.02% Citrox BCL for motility (panel (**B**)). Each point represents the mean ± standard deviation. The EPS results are expressed as percentage of the control.

The observed reduction in biofilm formation suggests that c-di-GMP production might regulate the EPS, hence inhibiting *L. pneumophila* biofilm. Our results show that EPS concentration decreased significantly during the growth of *L. pneumophila* in the presence of 0.02% Citrox BCL (Figure 4, panel A). We also show that the sub-inhibitory concentration of 0.02% induced a ~45% reduction in EPS production compared to the control and untreated *L. pneumophila* culture. The concentration of 0.01% was also used in this case to identify if further reductions are achieved; however, our data shows that below 0.02%, a plateau reduction is achieved. Further, we show that a significant reduction in motility was achieved by using the sub-inhibitory concentration of 0.02% (*p* = 0.002) (Figure 4, panel B). Taken together, these data suggest that the previously observed reduction in biofilm formation is also associated with reductions in two of the factors responsible for biofilm formation, EPS and motility, following exposure to 0.02% Citrox BCL.

### 3.5. Impact on Iron Sequestration and Siderophore Production

**Figure 5:**
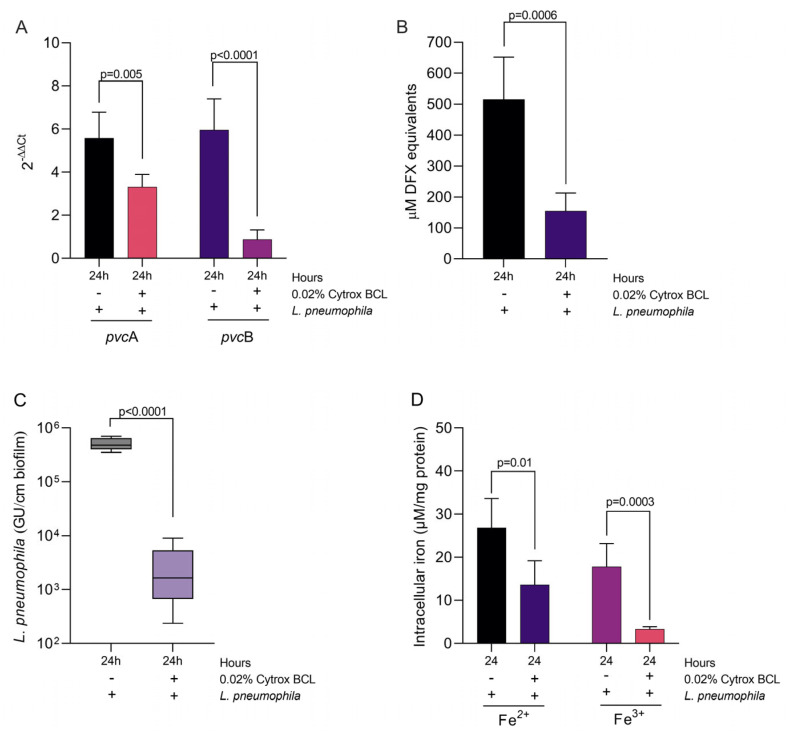
Expression of *pvc*A and *pvc*B genes (Panel (**A**)), CAS reactivity in supernatants (Panel (**B**)), number of *L. pneumophila* cells in formed biofilm (Panel (**C**)) and the levels of iron in formed biofilms (Panel (**D**)). The results are presented as means and standard deviations from triplicate samples and from three separate experiments. The supernatant’s CAS reactivity was tested in triplicates from three separate experiments (*p* < 0.05; Student’s *t*-test).

To further investigate the mechanisms involved in biofilm reduction, we have investigated the impact of Citrox BCL on iron sequestration genes *pvc*A and *pvc*B and siderophore activity. Our results show that the expression of *pvc*A and *pvc*B was significantly reduced (*p* = 0.005 and *p* < 0.0001) after 24 h in the presence of 0.02% Citrox BCL (Figure 5, panel A). The observed decrease in gene expression was also associated with a significant decrease in siderophore detection, in the culture supernatant, in the presence of 0.02% Citrox CBL (*p* = 0.0006) (Figure 5, panel B). The reduction in siderophore release was probably due to lower numbers of *L. pneumophila* cells (*p* < 0.0001) (Figure 5, panel C). This has caused decreased levels of Fe^2+^ and Iron ^3+^ detected in the formed biofilms in the presence of 0.02% Citrox BCL (Figure 5, panel D). These results suggest that Citrox CBL is involved in biofilm reduction by decreasing the ability of *L. pheumophila* to sequester iron required for growth and survival.

## 4. Discussion

Natural antimicrobials, such as essential oils, have been previously indicated as effective against *L. pneumophila* with great potential to be used as antimicrobials against this pathogen [33]. This study indicates that the spread of legionellosis in homes and resorts was successfully controlled with *Cinnamomum osmophloeum* leaf oil (cinnamaldehyde as a major constituent), having great potential to be used as an antibacterial agent in such circumstances. Their antimicrobial efficiency is improved when used as vapours in water, as it has been shown that citrus essential oil increased with 61% the presence of linalool. Therefore, establishing the proper delivery methods may dictate the level of efficiency [34].

Our study correlates the reduction in c-di-GMP production with the decrease in biofilm formation and significantly reduced EPS levels. Mixtures of organic acids have been previously shown to reduce EPS and biofilm production in *E. coli* O157:H7 [25]. EPS production is regulated by c-di-GMP, specifically of those involved in biofilm formation [35]. The down-regulation of the genetic factors, *tat*B and *tat*C, observed in our study is linked to reduced EPS levels detected, as they are part of the putative twin-arginine translocation pathway required for transmembrane protein translocation [36]. There is a clear connection between c-di-GMP and ROS tolerance [37]. Our data indeed shows that the ROS levels are reduced in infected and treated A549 cells, results which correlate with lower levels of c-di-GMP and lower infection rates. A link between iron regulation and c-di-GMP has been suggested as important in mediating biofilm formation in *P. aeruginosa* [38]. Biofilm formation in *L. pneumophila* is tightly controlled by iron as it is considered an essential nutrient during growth and replication [39] and mediates bacterial biofilm formation [31]. In *L. pneumophila,* iron concentration is controlled by the *pvc*A and *pvc*B genes which are involved in the production of siderophores and thus contribute to the uptake and sequestration of iron [8]. In our study, Citrox CBL was able to reduce siderophore levels and the expression of *pvc*A and *pvc*B genes, suggesting its involvement in bacterial iron starvation. Moreover, we show that the reduction in siderophore secretion, in the presence of Citrox CBL, is correlated with a decline in intracellular iron in biofilm-forming *L. pneumophila* cells. This observation clearly highlights and justifies the decrease in *L. pneumophila* virulence in A549 cells since iron bioavailability plays a fundamental role and regulates host-pathogen interactions [8].

Our results are in line with the current knowledge regarding biofilm regulation mechanisms in bacteria and novel regarding antimicrobial interference. For example, biofilm regulation factors are attributed to bacterial second messenger cyclic-dimeric diguanylate (c-di-GMP), where GGDEF/EAL-containing proteins were correlated with positive regulation of biofilm establishment by *L. pneumophila* [7]. For instance, *L. pneumophila* harbouring the absence of these proteins demonstrated a decreased biofilm formation, regardless of whether the expression of c-di-GMP was not different compared to the wild-type pathogen. Furthermore, the deletion of GGDEF/EAL proteins has been previously associated with the overproduction of biofilm, followed by a decrease in the expression of c-di-GMP [7]. Haem nitric oxide/Oxygen (H-NOX) is an additional binding domain of heme protein families involved in the c-di-GMP activity and biofilm regulation [7]. Compared to other prokaryotes, *L. pneumophila* is a unique prokaryote found to encode 2 H-NOX proteins in its genomes. The deletion of H-NOX-1 led to hyper biofilm formation without affecting the growth of the pathogen in murine macrophages, *Acanthamoeba castellanii* or proficient media [7]. Another cluster of genes orchestrated by *ahpC2* and *ahpD* encrypts alkyl hydroperoxide reductases associated with playing an influential role in the defence against oxidative stress in the formed biofilm cells [7]. Similarly, *FliA* responds to *L. pneumophila* biofilm formation, allowing the pathogen to thrive in unfavourable conditions [40]. A recent in vitro study showed that the sessile form of *L. pneumophila* displayed a phenotypic heterogeneity and was able to form non-growing and growing bacterial populations [41]. Specifically, the non-growing sessile cells showed high metabolic activity and could lead to a dormant phase by promoting additional long-term survivability in the environmental niche [41]. Moreover, the authors indicated that *LvbR* is a pleiotropic regulator corresponding to shape the biofilm architecture, pathogen-host cell relations and competence for DNA uptake.

## 5. Conclusions

**Figure 6:**
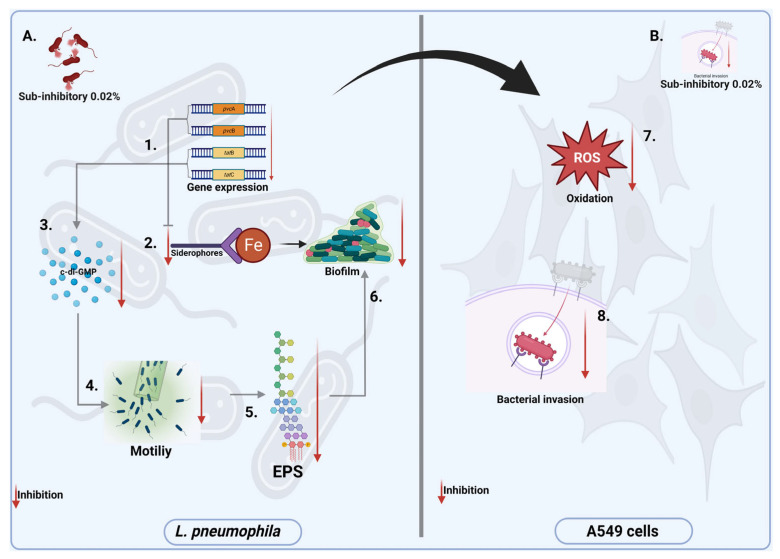
Summary of conclusions. (**A**) In *L. pneumophila* and (**B**) in A549 cells. Citrox BCL downregulates the *pvc*A and B gene expression leading to iron starvation in biofilms by lowering the production of siderophore molecules (1,2,3–6). Causes significant reduction in the production of c-di-GMP molecules (3) and downregulates *tat*B and C genes expression, reducing bacterial motility (4) and EPS production (5). Citrox BCL at sub-inhibitory concentrations (0.02%) reduces host oxidative stress (7) and inhibits bacterial invasion of A549 epithelial cells (8). Created with BioRender.com.

Our study shows that Citrox BCL inhibits a succession of biological events in *L. pneumophila* metabolism, which ultimately impact the bacterium’s ability to form biofilm and express virulence. First, it downregulates the *pvc*A and B gene expression leading to iron starvation in biofilms by lowering the production of siderophore molecules (Figure 6:1–6). Secondly, it causes a significant reduction in the production of c-di-GMP molecules (Figure 6:3) via the downregulation of the *tat*B and C genes which ultimately impact bacterial motility (Figure 6:4) and EPS production (Figure 6:5). All these events lead to reduced capacity to lower numbers of bacteria in biofilms and, therefore, reduced biofilm presence. Moreover, our study shows that the impact on all the above virulence factors of natural antimicrobials mixtures, such as Citrox BCL at sub-inhibitory concentrations (0.02%), will reduce host oxidative stress (Figure 6:7) and ultimately inhibit bacterial invasion of epithelial cells (Figure 6:8).

## Data Availability

Not applicable.
